# Research on Adaptive Discriminating Method of Brain–Computer Interface for Motor Imagination

**DOI:** 10.3390/brainsci15040412

**Published:** 2025-04-18

**Authors:** Jifeng Gong, Huitong Liu, Fang Duan, Yan Che, Zheng Yan

**Affiliations:** 1 College of Information Science and Engineering, Huaqiao University, Xiamen 361021, China; 22014082005@stu.hqu.edu.cn (J.G.); 22013082050@stu.hqu.edu.cn (H.L.); duan.fang@hqu.edu.cn (F.D.); 2 Engineering Research Center for Big Data Application in Private Health Medicine, Fujian Province University, Putian 351100, China

**Keywords:** motor imagery, brain–computer interface, feature extraction, adaptability, functional connectivity, electroencephalogram

## Abstract

(1) **Background**: Brain–computer interface (BCI) technology represents a cutting-edge field that integrates brain intelligence with machine intelligence. Unlike BCIs that rely on external stimuli, motor imagery-based BCIs (MI-BCIs) generate usable brain signals based on an individual’s imagination of specific motor actions. Due to the highly individualized nature of these signals, identifying individuals who are better suited for MI-BCI applications and improving its efficiency is critical. (2) **Methods**: This study collected four motor imagery tasks (left hand, right hand, foot, and tongue) from 50 healthy subjects and evaluated MI-BCI adaptability through classification accuracy. Functional networks were constructed using the weighted phase lag index (WPLI), and relevant graph theory parameters were calculated to explore the relationship between motor imagery adaptability and functional networks. (3) **Results**: Research has demonstrated a strong correlation between the network characteristics of tongue imagination and MI-BCI adaptability. Specifically, the nodal degree and characteristic path length in the right hemisphere were found to be significantly correlated with classification accuracy (*p* < 0.05). (4) **Conclusions**: The findings of this study offer new insights into the functional network mechanisms of motor imagery, suggesting that tongue imagination holds potential as a predictor of MI-BCI adaptability.

## 1. Introduction

The brain–computer interface (BCI) paradigm refers to the specific approaches and methods used in a BCI system to extract and decode brain signals. Common BCI paradigms include motor imagery, visual evoked potentials, event-related potentials, steady-state somatosensory evoked potentials, and others [1]. In the motor imagery-based BCI (MI-BCI) paradigm, subjects generate measurable brain signals by imagining specific limb movements. Processing these signals not only enables applications such as prosthetic limb control or cursor movement, but its applications also have expanded beyond healthcare to multiple areas of human–computer interaction [2,3]. That is, imagery of specific tasks that include hand, foot, and tongue movements systematically mobilizes the primary motor cortex and activates some body part-specific representations in nonprimary motor areas [4]. The advantage of MI-BCI is that it does not require external stimuli (unlike reactive BCI), and it relies on extensive training to enable users to generate ideal brain activity patterns for the system to classify [5,6].

In particular, motor imagery was also characterized inconsistently across different EEG signal rhythms. Giromini et al. showed that significant mu-band suppression occurs when subjects perceive motion, regardless of the experimental conditions [7]. Giacomo et al. found significant changes in α and beta power in prefrontal areas during a motor imagery task of the hand [8]. Furthermore, Cattai et al. indicated that network characteristics in the sensorimotor regions, based on spectral coherence, are enhanced during motor imagery [9]. The theoretical basis for the generation of motor imagery signals is event-related desynchronization (ERD) and event-related synchronization (ERS) [10]. Giacomo et al. found that during a motor imagery task, the α (8–13 Hz) and beta (13–30 Hz) wave power in the prefrontal regions of the brain of the subjects varied significantly, and that, in particular, the α wave power usually showed a trend towards event-related synchronization during motor imagery phenomenon, whereas beta-wave power showed a tendency towards event-related synchronization [8]. It is important to quantify as much as possible the results in each specific frequency range in a motor imagery task.

However, approximately 37.5% of healthy subjects have a classification accuracy below 70% in MI-BCI tasks, indicating significant differences in BCI adaptability among individuals [11]. Additionally, Phunruangsakao et al. pointed out that some participants require extensive training, which may reduce the efficiency of BCI applications for certain individuals [12]. Different brain regions are functionally specific, and different limbs correspond to different sensorimotor regions of the cortex [13]. To effectively select individuals suitable for MI-BCI technology, a deeper understanding of the functional characteristics of different brain regions during motor imagery tasks is necessary [14,15]. The need to screen individuals who are suitable for MI-BCI technology requires a deeper understanding of the functional characteristics of different brain regions during the execution of motor imagery tasks.

Functional connectivity analysis can be used as a tool to examine the interactions between brain regions during motor imagery tasks [16]. Identifying which brain regions play key roles in motor imagery helps in understanding the neural mechanisms of motor control [17]. Functional connectivity is interpreted as the statistical dependencies between brain regions, while effective connectivity provides directionality to this information exchange [18,19]. Hamedi M et al. mentioned in their research that functional connectivity analysis, particularly functional and effective connectivity, has been described as one of the most promising approaches to improve MI-BCI performance [16]. Feng Z studied the performance of functional connectivity features, measured by Phase Lag Index (PLI), Weighted Phase Lag Index (WPLI), and Phase Locking Value (PLV), in motor imagery classification, demonstrating that functional connectivity features can serve as powerful tools for identifying different motor intentions [20]. Since complex brain networks presented by high-density EEG are not easy to analyze, describing brain connectivity patterns based on graph theory possesses high efficiency [21]. Much of the previous research has been limited to the role of functional connectivity traits in enhancing MI-BCI performance and has not explored the association of important brain connections with subjects’ adaptive capacity in the context of this role.

In this study, we designed a motor imagery experiment with 50 subjects, recording electroencephalogram (EEG) activity during four distinct motor imagery tasks, with a focus on the 8–30 Hz frequency band. Motor imagery classification accuracy was used to quantify subjects’ MI-BCI adaptability. We calculated functional connectivity during the imagery tasks using WPLI and computed network properties based on graph theory. Finally, we explored the correlation between MI-BCI adaptability and brain network characteristics to identify key indicators of motor imagery.

## 2. Materials and Methods

### 2.1. Participants

The experiment involved 50 participants (aged 20 to 25 years and right-handed). All participants had no sensory or cognitive impairments, and their vision was either normal or corrected to normal. Each participant signed an informed consent form before the experiment. The protocol was approved by the Ethics Committee of Huaqiao University.

### 2.2. Experimental Data Collection and Processing

#### 2.2.1. Experimental Data Collection

Before participants underwent formal data collection, a video of the prescribed movements for motor imagery was viewed to ensure that subjects imagined the movements as consistently as possible. The details of the specific movements for motor imagery were lifting the left hand, lifting the right hand, lifting both feet at the same time, and sticking out the tongue. Participants had no prior exposure to motor imagery tasks or the BCI system.

**Figure 1:**
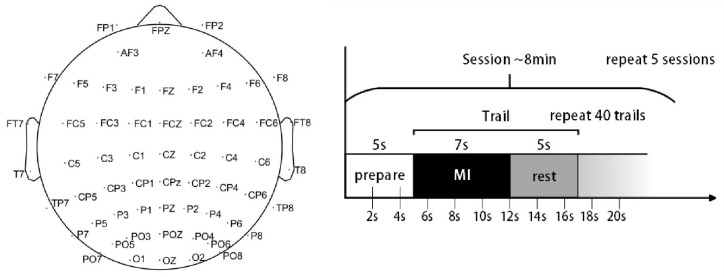
Experimental paradigm.

The experimental paradigm included four types of motor imagery tasks (left hand, right hand, foot, and tongue). In Figure 1, participants were prompted to imagine the movement for 7 s, followed by a rest prompt, and then a 5 s rest period before the next imagery task. One imagery task followed by one rest period constituted one trial. Each set consisted of 40 trials (each of the four imagery tasks appearing randomly 10 times), and participants completed five sets in total. Thus, each participant performed 200 trials, with each type of imagery task being performed 50 times. The data acquisition equipment used was a Neuro Scan, with electrodes arranged according to the international 10–20 system and a sampling rate of 1000 Hz.

#### 2.2.2. Data Processing

This preprocessing is performed in the EEGLAB version 2022.1 and includes the following steps [22]: (1) Electrode matching: electrode positioning, i.e., matching of electrode spatial coordinate positions, was performed before data preprocessing to obtain the positioning coordinates of each electrode. (2) Electrode picking and interpolation: electrodes not used in this study were removed, and interpolation operations were performed on the bad guides. Sixty electrodes were retained for this study, specifically FP1, FPZ, FP2, AF3, AF4, F7, F5, F3, F1, FZ, F2, F4, F6, F8, FT7, FC5, FC3, FC1, FCZ, FC2, FC4, FC6, FT8, T7, C5, C3, C1, CZ, C2, C4, C6, T8, TP7, CP5, CP3, CP1, CPZ, CP2, CP4, CP6, TP8, P7, P5, P3, P1, PZ, P2, P4, P6, P8, PO7, PO5, PO3, POZ, PO4, PO6, PO8, O1, OZ, and O2. (3) Re-referencing: The whole electrode averaging reference method was used. (4) Downsampling: To reduce the complexity of the data and the time for subsequent calculations, the data were downsampled to 250 Hz. (5) Filtering: A finite impulse response filter (FIR) was used with a filter order of three times the ratio of the data sampling rate to the transition bandwidth, a cutoff transition bandwidth of 1 Hz was set to ensure the steepness of the filter, and a Hanning window was used to minimize the side-valve effect. High-frequency and low-frequency noises often occur during EEG acquisition, and the data were band-pass filtered using an FIR filter from 1 to 40 Hz to remove the low-frequency and high-frequency noises. The filter range includes EEG bands related to motor imagery, such as α-band (8–13 Hz) and β-band (13–30 Hz). (6) Segmentation: The motor imagery epoch of each subject was extracted, and the dimension of the integrated data was 50 × 750 × 35 × 4 (the first dimension represented 50 people, the second dimension represented 3 s of imagery taken, the third dimension represented 35 valuable trainings per imagery by manually filtering, and the fourth dimension represented 4 kinds of motor imagery tasks). (7) Independent Component Analysis (ICA): ICA is performed to categorize each independent component obtained from ICA into noise and non-noise by examining the time-domain waveform and power spectral density. After eliminating the noise components, the noise-free data will be applied to subsequent stages.

### 2.3. MI-BCI Adaptability

The adaptability of the MI-BCI in this study was quantified by measuring the classification accuracy of motor imagery tasks, specifically the ability of participants to correctly classify different motor imagery tasks. Pairwise classification was performed between motor imagery tasks, and each participant received six classification results (left hand–right hand, left hand–foot, left hand–tongue, right hand–foot, right hand–tongue, and foot–tongue). The average of these six classification results was used as an index to quantify each participant’s MI-BCI adaptability. To ensure the reliability of the results, classification accuracy was calculated using four different feature extraction methods.

### 2.4. Feature Extraction and Classification

#### 2.4.1. Power Spectral Density

Power spectral density (PSD) is a tool used to represent the power distribution of a signal in the frequency domain, particularly suitable for analyzing non-stationary signals like EEG [23]. This study employed the multi-taper method for PSD, which provides a more accurate spectral representation compared to the traditional Welch method. PSD was computed in the frequency range of [8, 30 Hz] to extract features.

#### 2.4.2. Wavelet Transform and Common Spatial Pattern

This method utilized discrete wavelet transform (DWT) and wavelet packet decomposition (WPD) techniques to extract time–frequency features [24]. At each decomposition level, high-pass and low-pass filters were applied. Appropriate mother wavelets, such as Daubechies, Symlets, Haar, and Coiflets series, were selected based on the characteristics of each participant’s EEG data. DWT was set to five decomposition levels, while WPD was set to three, producing multiple sub-bands. The average power, mean, and standard deviation of each sub-band were calculated, and the coefficients of all sub-bands were used as features. The common spatial pattern (CSP) algorithm was further applied to merge sub-band features, enhancing feature representation [25].

#### 2.4.3. Riemannian Manifold

This method first decomposed the preprocessed signals into six different frequency bandpass-filtered signals, calculating the covariance matrix for each band. The Riemannian mean was computed to determine the central position of each covariance matrix. Each covariance matrix was then logarithmically mapped to the tangent space of the Riemannian manifold (RF), transforming nonlinear covariance matrices into symmetric matrices in linear space [26]. The log-mapped results in the tangent space were used as feature vectors.

#### 2.4.4. Filter Bank Common Spatial Pattern

In the filter bank CSP (FBCSP) method, the preprocessed signals were decomposed into multiple frequency bandpass-filtered signals, and the CSP algorithm was applied to each band to extract features [27]. The best-performing bands were selected as features using mutual information methods. This approach, by processing multiple bands, flexibly adapts to different participants’ brain activity patterns, thus improving the BCI system’s recognition ability.

#### 2.4.5. Classification

The aim of this study was not to develop a classification method but rather to explore individual differences in the adaptability of MI-BCI. In this case, the choice of SVM was motivated by its broader applicability and reliability in motor-related EEG decoding. Therefore, in this study, SVM is used for motion image classification [28,29,30]. Four feature extraction methods were used as the original feature set, and a 10-fold cross-validation strategy was employed to evaluate the performance of the classifier model in terms of accuracy. The four feature extraction methods are described next.

### 2.5. Brain Functional Network Construction

#### 2.5.1. Functional Connection

Brain functional networks are crucial tools for assessing the connectivity relationships between various nodes or regions of the brain. During cognitive activities related to motor imagery, the associated brain functional networks exhibit significant changes, reflected in the adjustments of network structures. In high-density EEGs, WPLI performs relatively well in reducing the sensitivity to additional uncorrelated noise sources, as well as improving the statistical ability to detect phase synchronization changes [31]. In this study, we constructed brain networks by calculating the weighted phase lag index (WPLI) [32] and plotted and analyzed motor imagery-related functional connectivity maps in the α band (8–12 Hz), β1 band (13–20 Hz), and β2 band (20–30 Hz). During this process, 60 EEG electrode points were selected to generate a 60 × 60 functional connectivity matrix. To clearly display the active brain region nodes during motor imagery, the top 10% of the most significant connections were chosen as the threshold for further analysis. WPLI is defined as follows:

(1) $${WPLI}_{xy}=\frac{\left|\frac{1}{N}\sum_{n=1}^{N}\left|im\left(S_{xy}^{n}\right)\right|sign(im\left(S_{xy}^{n}\right))\right|}{\frac{1}{N}\sum_{n=1}^{N}\left|im\left(S_{xy}^{n}\right)\right|}$$

where x and y are the EEG signals of the two channels, respectively; sign(⋅) represents the sign function; $\;S_{xy}$ represents the cross-power spectrum of x and y; and |⋅| represents the absolute value.

#### 2.5.2. Network Properties

Complex network analysis provides a method for describing and analyzing the intricate brain functional networks constructed based on motor imagery tasks. This analysis reveals the dynamic changes and characteristics of brain functional networks under different task states. To gain a deeper understanding of the impact of motor imagery tasks on brain functional networks, we explored several key network topology properties [33]. Detailed information is shown in Table 1.

## 3. Results

### 3.1. MI-BCI Adaptability Results

**Figure 2:**
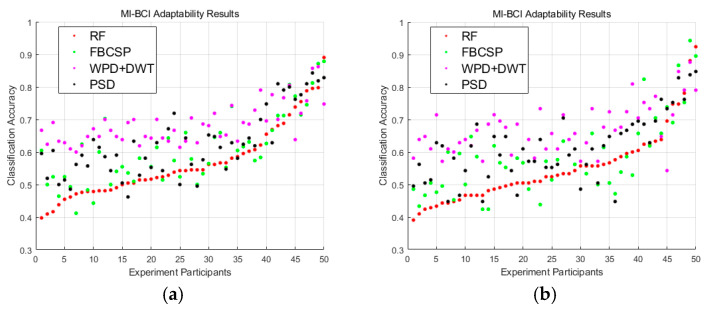
Participants were sorted by RF classification accuracy from smallest to largest, with a consistent total number of imagery counts per subject. (**a**) The average classification accuracy results for four classification methods corresponding to six motor imagery tasks (left hand vs. right hand, left hand vs. foot, left hand vs. tongue, right hand vs. foot, right hand vs. tongue, and foot vs. tongue) for each subject. (**b**) The average classification accuracy results for three classification methods corresponding to three motor imagery tasks (left hand vs. right hand, left hand vs. foot, and right hand vs. foot) for each subject.

This study involved 50 participants, and their MI-BCI adaptability was evaluated using four different classification methods. Since the number of each motion imagery is 35, the sample size is clarified to be 70 before performing the binary classification. 21 channels are selected to compute the mean PSD feature, and the data dimension is 70 × 21. Six sub-bands are selected using DWT and WPD, and the mean and standard deviation of the wavelet decomposition coefficients and the mean PSD are computed as fusion features for each sub-band, and the data dimension is 70 × 36. Data are divided into 6 sub-bands, and 21 channels are selected, two Riemann distance features for each sub-band in two types of Riemann mean (21 × 21 surface), and the data dimension is 70 × 12. FBCSP is applied to six sub-bands of EEG signals to extract the spatial information, and two sets of spatial filters are extracted to produce four features, and the data dimension is 70 × 24. The above feature matrix is input into the SVM for classification. As shown in Figure 2, the adaptability of each participant was calculated as the average pairwise classification accuracy across motor imagery tasks.

**Figure 3:**
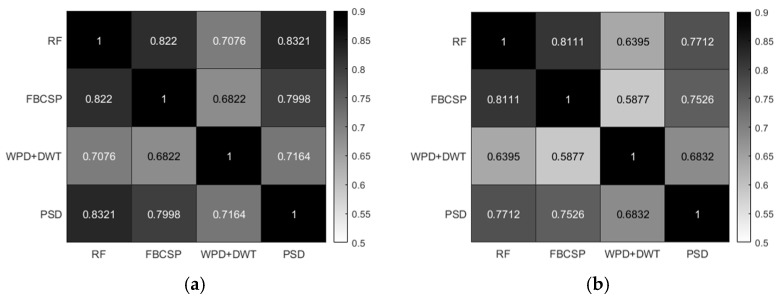
(**a**) Pearson correlation analysis results of the average classification accuracies across four classification methods for six motor imagery tasks (left hand vs. right hand, left hand vs. foot, left hand vs. tongue, right hand vs. foot, right hand vs. tongue, and foot vs. tongue). (**b**) Pearson correlation analysis results of the average classification accuracies across three classification methods for three motor imagery tasks (left hand vs. right hand, left hand vs. foot, and right hand vs. foot).

To ensure consistency in classification results across all participants, Pearson correlation analysis was conducted on the results of different methods [34]. As shown in Figure 3, all correlation results demonstrated a significant positive correlation (*p* < 0.001), indicating a high degree of consistency among different classification methods. This result confirms the validity of each classification method and provides evidence that they reliably reflect participants’ MI-BCI adaptability.

### 3.2. Brain Network Visualization Results

**Figure 4:**
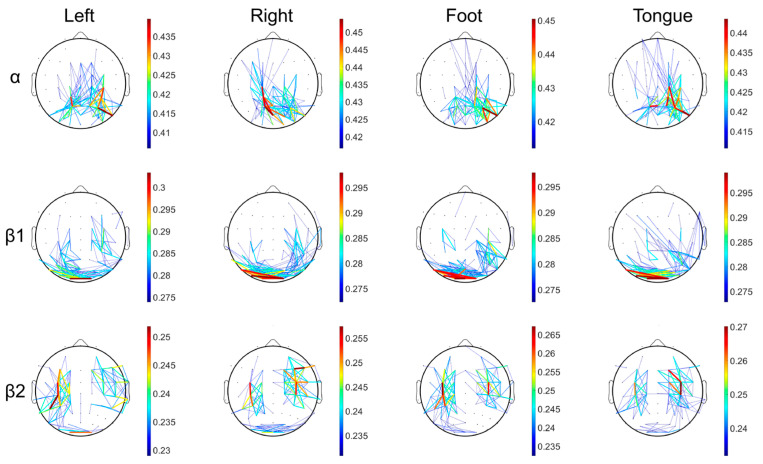
As shown in the figure, the brain network connectivity strength for the four imagery tasks is displayed. The first row represents the α band, the second row the β1 band, and the third row the β2 band, with the threshold set to the top 10% of connectivity strength.

Figure 4 shows the brain network visualization results for the four motor imagery tasks (left hand, right hand, foot, and tongue) in the α band, β1 band, and β2 band. In the β2 band, the left hand and right hand imagery tasks exhibited significant contralateral inhibition, meaning the brain activity on one side showed an inhibitory response on the opposite side. Conversely, in the α band, left hand and right hand imagery showed ipsilateral inhibition, indicating that the brain region on the active side exhibited an inhibitory response on the same side. Notably, the results for the tongue motor imagery task showed distinct functional differences between the left and right brain regions, and the connectivity strength was mainly concentrated in the central, frontal, and parietal regions.

### 3.3. Correlation Analysis Results Between Brain Networks and MI-BCI Adaptability

This study, through the analysis of brain network visualization results from 50 subjects, identified the primary points of connectivity strength across 15 electrodes in each hemisphere of the brain. The key electrodes in the left hemisphere include F5, F3, F1, FC5, FC3, FC1, C5, C3, C1, CP5, CP3, CP1, P5, P3, and P1, while in the right hemisphere, the key electrodes include F6, F4, F2, FC6, FC4, FC2, C6, C4, C2, CP6, CP4, CP2, P6, P4, and P2. A statistical analysis of the network properties of these electrodes was conducted, including degree (K), clustering coefficient (C), characteristic path length (L), local efficiency (E), and betweenness centrality (BC). The average network properties for the left and right hemispheres were denoted as KL, CL, LL, EL, and BCL (left hemisphere) and KR, CR, LR, ER, and BCR (right hemisphere), respectively. Additionally, the characteristic path length for all 30 electrodes in both hemispheres was calculated and denoted as L. Given the significant differences in connectivity strength and network properties between the hemispheres, this study further computed the difference values of these network properties between the left and right hemispheres, denoted as degree difference (KD), clustering coefficient difference (CD), characteristic path length difference (LD), local efficiency difference (ED), and betweenness centrality difference (BCD).

**Figure 5:**
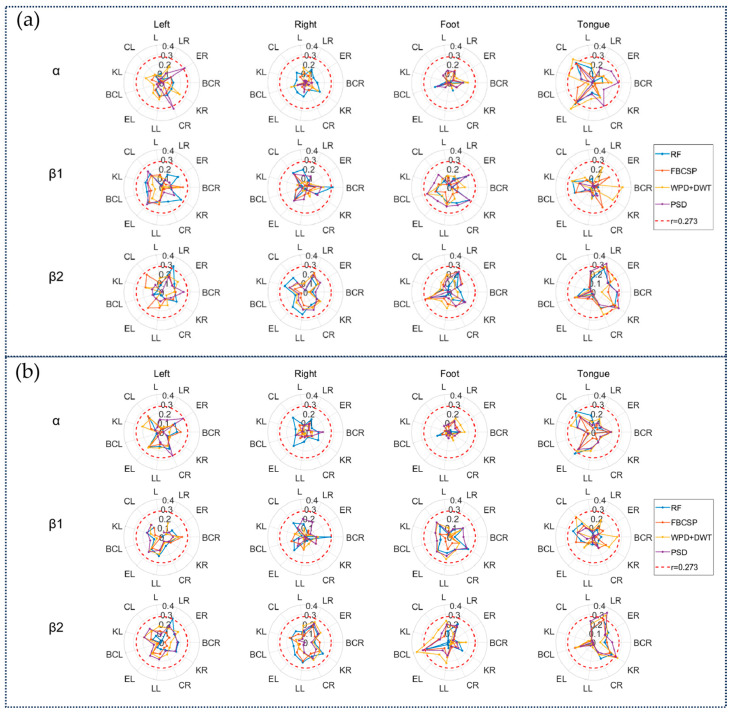
(**a**) The correlation results between the imagined adaptability (represented by four classification results: left hand vs. right hand, left hand vs. foot, left hand vs. tongue, right hand vs. foot, right hand vs. tongue, and foot vs. tongue) and the network properties of four motor imagery tasks. (**b**) The correlation results between the imagined adaptability (represented by three classification results: left hand vs. right hand, left hand vs. foot, and right hand vs. foot) and the network properties of four motor imagery tasks. Correlations highlighted in red circles indicate significant results (*p* < 0.05, r > 0.273).

We analyzed the correlation between these network properties and the MI-BCI classification results of 50 subjects using the Pearson correlation coefficient to evaluate the association between network properties and MI-BCI adaptability. As shown in Figure 5a, the motor imagery adaptability in the α band exhibited significant correlations with the left-hemisphere network properties of tongue imagery, while the β2 band primarily focused on the right hemisphere. As depicted in Figure 5b, this phenomenon persisted even when removing the tongue imagery task. Table 2 and Table 3 list the correlation results for motor imagery adaptability with tongue imagery tasks in the α and β2 bands, respectively.

## 4. Discussion

The findings indicated that right brain network properties in the β2 frequency band of the tongue imagery task were positively correlated with MI-BCI adaptation (*p* < 0.05). This finding can be explained by the functional differentiation between the left and right hemispheres of the brain. The right brain is dominant in processing spatial perception, holistic pattern recognition, and emotion processing [35], and tongue imagery may activate right brain networks associated with spatial localization and proprioception. For example, tongue movements require precise spatial perception to coordinate complex movements in the oral cavity, and this perception may rely on the right brain’s parietal-frontal network [36]. The high connectivity strength in the β2 band may reflect the right brain’s high efficiency in integrating multimodal sensory-motor information, which is precisely what makes the MI-BCI system so efficient at decoding complex motor intentions. In contrast, suppression in the α band usually reflects disinhibition of the relevant brain region [36]. This provides important clues to our interpretation of connectivity differences in the tongue imagery task. The functional differences between the left and right hemispheres became important for predicting MI-BCI adaptability [35].

Whereas tongue imagery evokes stronger right hemisphere connectivity compared to hand and foot tasks, i.e., the correlation between hand and foot imagery tasks is weaker. This phenomenon may be related to the neuroanatomical basis of tongue movements. Compared to limb movements, tongue movements involve more complex bilateral synergistic activation of the sensorimotor cortex, especially in the cross-projecting regions of the precentral and postcentral gyrus [36]. This bilateral synergistic activation may have enhanced functional connectivity across the hemispheres, thus showing higher network feature stability in the β2 frequency band. In contrast, hand and foot imagery primarily activates the unilateral motor cortex, whose functional networks may be more susceptible to interindividual differences in neuroplasticity. Studies have found that tongue motor imagery not only activates motor representations but also engages somatosensory representations related to the movement [36,37].

The tongue imagery task showed significant predictability at the group level; there were still differences in adaptation between individuals. Such differences may stem from the dynamic plasticity of neural networks. For example, individuals with long-term fine-motor training (e.g., musicians or surgeons) may exhibit stronger right-brain β2-band connectivity strength, whereas individuals lacking such training may require longer BCI calibration [12]. Future studies could verify the causal relationship between neural network plasticity and MI-BCI adaptation through longitudinal experiments, for example, by comparing changes in brain network properties before and after training.

Although this study verified the reliability of the results through multiple feature extraction methods, the functional connectivity analysis mainly relied on the WPLI. Future studies could introduce other connectivity metrics and dynamic functional connectivity methods for the analysis. In addition, the current experiments focused only on healthy subjects, whereas the core user groups of BCI technology (e.g., paralyzed patients) may exhibit different neural network patterns. Future studies should be extended to clinical populations to verify the generalizability of tongue imagery as a predictor.

## 5. Conclusions

Through detailed experimental design and data analysis, this study investigated the adaptability of MI-BCI and its relationship with brain functional network properties. The results demonstrated a strong correlation between the network properties of the right hemisphere during tongue imagery tasks in the β2 band and MI-BCI adaptability. Additionally, this study showed that precise analysis of network attributes such as node degree and characteristic path length can better predict individual performance in MI-BCI systems. Analyzing brain networks can more accurately reveal the role of brain functional networks in motor imagery tasks, thereby providing a basis for optimizing the design and personalization of MI-BCI systems. Furthermore, the methods proposed in this study offer an effective screening strategy for initially identifying suitable MI-BCI users, helping to recognize individuals most likely to benefit from BCI training.

## Figures and Tables

**Table 1:** Specific computational methods for network properties extraction.

Method	Calculation Formula	Remark
Node degree	$k_{i}=\sum_{j=1}^{N}A_{ij}$	N represents the total number of nodes, $A_{ij}$$\;\mathrm{represents}\;\mathrm{the}\;\mathrm{element}\;\mathrm{of}\;\mathrm{the}\;\mathrm{adjacency}\;\mathrm{matrix},\;k_{i}$ represents the degree of node i, $e_{i}$ represents the number of edges between the neighbors of node i, $d_{ij}$ represents the shortest path length between node i and node j, $\sigma_{st}(v)$ represents the number of shortest paths from node s to node t that pass through node v.
Clustering coefficient	$C_{i}=\frac{2e_{i}}{k_{i}(k_{i}-1)}$	
Characteristic path length	$L=\frac{1}{N(N-1)}\sum_{i\neq j}d_{ij}$	
Local efficiency	$E_{local}\left(v\right)=\frac{1}{N_{i}(N_{i}-1)}\sum_{i\neq j}\frac{1}{d_{ij}}$	
Betweenness centrality	${BC}_{v}=\sum_{s\neq v\neq t}\frac{\sigma_{st}(v)}{\sigma_{st}}$	

**Table 2:** The correlation analysis results of tongue imagery in the α band.

	RF		FBCSP		WPD + DWT		PSD	
	*p*	r	*p*	r	*p*	r	*p*	r
CL	0.050 *	0.278 *	0.060	0.267	0.019 *	0.330 *	0.143	0.210
KL	0.371	0.129	0.691	0.058	0.060	0.268	0.339	0.138
BCL	0.983	0.003	0.236	−0.171	0.700	−0.056	0.953	0.008
EL	0.037 *	0.297 *	0.046 *	0.283 *	0.009 *	0.364 *	0.075	0.254
LL	0.461	0.107	0.799	0.037	0.228	0.174	0.405	0.120
CD	0.049 *	−0.280 *	0.042 *	−0.288 *	0.021 *	−0.326 *	0.025 *	−0.316 *
KD	0.630	−0.070	0.756	−0.045	0.304	−0.148	0.272	−0.158
BCD	0.646	−0.066	0.941	0.011	0.535	−0.090	0.216	−0.178
ED	0.077	−0.253	0.071	−0.258	0.032 *	−0.304 *	0.032 *	−0.304 *
LD	0.608	−0.074	0.618	−0.072	0.291	−0.152	0.208	−0.181

* *p* < 0.05 and r > 0.273 indicate that the network attributes corresponding to the values are significantly correlated with the corresponding accuracy methods.

**Table 3:** The correlation analysis results of tongue imagery in the β2 band.

	RF		FBCSP		WPD + DWT		PSD	
	*p*	r	*p*	r	*p*	r	*p*	r
CR	0.198	0.185	0.177	0.194	0.066	0.262	0.312	0.146
KR	0.061	0.267	0.077	0.252	0.033 *	0.303 *	0.024 *	0.320 *
BCR	0.079	0.251	0.278	0.156	0.535	0.090	0.075	0.254
ER	0.194	0.187	0.137	0.213	0.059	0.269	0.218	0.177
LR	0.056	0.272	0.018 *	0.332 *	0.037 *	0.296 *	0.018 *	0.332 *
CD	0.125	0.220	0.387	0.125	0.155	0.204	0.527	0.092
KD	0.088	0.244	0.274	0.158	0.096	0.238	0.104	0.233
BCD	0.033 *	0.302 *	0.085	0.246	0.186	0.190	0.047 *	0.282 *
ED	0.153	0.205	0.398	0.122	0.117	0.224	0.450	0.109
LD	0.073	0.256	0.148	0.208	0.064	0.264	0.081	0.249

* *p* < 0.05 and r > 0.273.

## Data Availability

The data supporting this study’s findings are available on request from the corresponding authors, Z.Y. However, the data are not publicly available because they contain information that could compromise the privacy of research participants.
